# A comparison of five different drug-drug interaction checkers for selective serotonin reuptake inhibitors

**DOI:** 10.3389/fphar.2025.1690975

**Published:** 2025-09-24

**Authors:** Shanshan Xu, Zhihui Song, Yiman Li, Jie Bai, Dong Wang, Ente Wang, Jiawei Wang

**Affiliations:** Department of Pharmacy, Beijing Tongren Hospital, Capital Medical University, Beijing, China

**Keywords:** drug-drug interaction checkers, selective serotonin reuptake inhibitors, potential drug-drug interactions, consistency evaluation, clinical decision-making

## Abstract

**Background:**

Selective serotonin reuptake inhibitors (SSRIs) are widely prescribed for depression and anxiety, but their potential for drug-drug interactions (DDIs) poses significant risks, particularly given their influence on cytochrome P450 enzymes. Variability in identifying and classifying these interactions among drug interaction checkers (ICs) can complicate clinical decision-making and compromise patient safety. This study aims to compare five widely used ICs in identifying DDIs related to SSRIs, highlighting discrepancies in DDI identification and severity classification to inform best practices.

**Methods:**

A comparative study was conducted using five popular ICs (Micromedex, Lexi-Interact, Epocrates, Medscape, and Drugs.com) to evaluate their performance in identifying SSRIs-related DDIs. Data on drug-SSRIs interaction pairs were extracted over 2 weeks in 2025. Descriptive analysis was used to quantify potential interactions and their severity. Gwet’s AC1 coefficient was employed to assess agreement among all five ICs and to compare groups of four- and two-pair sets of ICs.

**Results:**

A total of 1,190 potentially interacting drugs with fluoxetine (FXT) were reported, 1,129 for fluvoxamine (FVM), 1,131 for citalopram (CIT), 1,084 for paroxetine (PAR), 1,206 for sertraline (SER) and 1,146 for escitalopram (ESC). The agreement among all five ICs was notably low, with Gwet’s AC1 values ranging from 0.16 to 0.24 across different SSRIs. Similarly, it was poor in 4 and 2 sets analyses. The level of agreement among the ICs in classifying the severity of potential DDIs or restricting DDIs identified as severe was poor, also in 4 and 2 sets analysis.

**Conclusion:**

The findings reveal substantial discrepancies in the identification and severity categorization of SSRIs-related DDIs among ICs, underscoring the challenges faced by healthcare providers in ensuring safe prescribing practices. The study advocates for the standardization of IC databases and severity criteria to enhance consistency and reliability.

## 1 Introduction

Selective Serotonin Reuptake Inhibitors (SSRIs) hold a pivotal role in the pharmacological treatment of depression and anxiety disorders, impacting millions of individuals globally (Lochmann and Richardson, 2019; American Psychological Association, 2019; NICE, 2022). As a cornerstone of psychiatric medication, SSRIs, including fluoxetine (FXT), fluvoxamine (FVM), citalopram (CIT), paroxetine (PAR), sertraline (SER) and escitalopram (ESC), are frequently prescribed due to their efficacy and relatively favourable side effect profile compared to older antidepressants. SSRIs influence the activity of several cytochrome 450 isoenzymes, such as CYP3A4, 2D6, 2C9 and 2C19 (Margolis et al., 2000; Dobrea et al., 2025; Sangkuhl et al., 2011; von Moltke et al., 2001). Potential drug-drug interactions (DDIs) may occur when antidepressants are administered concomitantly with other pharmacological treatments. SSRIs-related DDIs can lead to serious adverse effects, such as decreased effectiveness, central nervous system (CNS) depression, neurotoxicity, QT-interval prolongation, and serotonin syndrome (Khan et al., 2019; Nguyen et al., 2020). The risk of SSRIs-related DDIs is particularly relevant because patients with depression are often treated with numerous concurrent medications (Ereshefsky et al., 2005). As such, healthcare providers rely on drug-drug interaction checkers (ICs) to navigate the complex landscape of polypharmacy, ensuring patient safety and optimizing treatment outcomes.

ICs are specialized software tools designed to identify potential interactions between medications. By providing healthcare professionals with crucial information, these tools play an essential role in clinical decision-making processes. Variability in the databases, algorithms, and criteria used by different ICs often leads to discrepancies in DDI identification and severity classification (Günay et al., 2022; Muhič et al., 2017). The variability in the identification and classification of DDIs by different ICs presents a significant challenge for healthcare providers. This inconsistency can lead to varying clinical outcomes, which may compromise patient safety and treatment efficacy. Given these issues, there is a pressing need for comparative studies that evaluate the effectiveness and reliability of various ICs in identifying DDIs, particularly those related to commonly prescribed drug classes such as SSRIs. By systematically examining the performance of different ICs, researchers can identify strengths and weaknesses in their design and implementation, ultimately contributing to improvements in their functionality and clinical utility.

The study aims to address these gaps by conducting a comparative analysis of five widely used ICs, evaluating their performance in identifying DDIs with SSRIs. This research seeks to provide a clearer understanding of the variability in DDI identification and severity classification among different checkers, thereby informing best practices in clinical decision-making.

## 2 Methods

### 2.1 Study design and data sources

A comparative study regarding the drug interactions of SSRIs was conducted using five ICs. The ICs were selected based on their popularity worldwide, as reviewed in the previous studies, including Micromedex Drug Interactions, IC 1; Lexi-Interact, IC 2; Epocrates, IC 3; Medscape, IC 4; and drugs.com, IC 5. Each IC is generally updated on a weekly, monthly or quarterly basis. In this study, data concerning all drug-SSRIs interaction pairs were extracted from the 5 ICs in the period between July 20 and 4 August 2025.

Each medication being studied is accompanied by all five information centers, which furnish an extensive inventory of possible interacting drugs along with in-depth details on the mechanisms of these potential DDIs and their respective severity levels. Although all centers categorize DDI severity with similar groupings, they employ distinct labels. To address this, the severity classifications from various sources were unified into four standardized groups: severe (meaning contraindicated or major DDIs), moderate, minor, and unknown (Supplementary Table S1).

We included SSRIs currently approved for use by the FDA. They are fluoxetine (FXT), fluvoxamine (FVM), citalopram (CIT), paroxetine (PAR), escitalopram (ESC), and sertraline (SER). No ethical approval or informed consent was required because this research did not involve human participants or animals.

### 2.2 Statistical analysis

First, a descriptive analysis was conducted to determine the number of drugs potentially interacting with each SSRI, as identified by each IC, and to illustrate the distribution of these drugs across different DDI severity levels assigned by each IC. Specifically, matrices were employed to display the proportion of potentially interacting drugs identified by each IC for each SSRI, while bar charts depicted the distribution of DDI severity categories. Second, the agreement level among the five ICs regarding the identification of potentially interacting drugs with each SSRI (a binary variable: interaction or non-interaction) was assessed by calculating Gwet agreement coefficients (AC1 values) and their 95% CIs (Gwet, 2014), which provides a measure of interrater reliability adjusted for chance, offering a reliable alternative to the kappa (κ) statistic, especially useful in cases of significant category imbalances, such as the severity levels in our study (Wongpakaran et al., 2013; Gisev et al., 2013). The Gwet AC1 coefficient interprets agreement as follows: a value of 1 signifies perfect agreement, 0.76 to 1 indicates excellent concordance, 0.41 to 0.75 suggests moderate to good agreement, and 0 to 0.40 signifies poor agreement. Negative values reflect disagreement, with −1 indicating complete disagreement. Values near zero with a nonsignificant P value suggest agreement indistinguishable from chance (Gwet, 2014). The same approach was used for comparisons among severe category DDIs for all 5 ICs (including only potential DDIs that were categorized as severe by at least 1 IC), as well as comparing groups of four ICs (by excluding one of the five ICs), three ICs (by excluding two of the five ICs) and among pairs of ICs (by excluding three of the five ICs). All analyses utilized R version 4.3.0 with specific statistical packages (irrCAC, irr, psych, and fmsb).

## 3 Results

The total number of unique DDIs identified across all five ICs after removing duplicates was: 1,190 with FXT, 1,129 with FVM, 1,131 with CIT, 1,084 with PAR, 1,206 with SER, and 1,146 with ESC (Table 1). IC 5 reported the most potential drug interactions across most SSRIs, followed by ICs 2, 3, 1, and 4 (Table 1). Notably, IC 5 accounted for over 54% of the total potential interactions for each SSRI, excluding PAR.

**TABLE 1:** Number of potentially interacting drugs identified by 5 ICs, stratified by SSRIs and DDI severity.

Interactions	IC, No. (% of total)					Total, No.
	IC 1	IC 2	IC 3	IC 4	IC 5	
FXT						
Potentially interacting drugs	363 (30.5)	647 (54.37)	567 (47.65)	403 (33.87)	705 (59.24)	1,190
Severe DDI	335 (74.78)	84 (18.75)	152 (33.93)	125 (27.90)	135 (30.13)	448
Moderate DDI	28 (2.94)	370 (38.82)	283 (29.70)	278 (29.17)	542 (56.87)	953
Minor DDI	0	193 (63.28)	132 (43.28)	0	28 (9.18)	305
FVM						
Potentially interacting drugs	475 (42.07)	632 (55.98)	570 (50.49)	404 (35.78)	661 (58.55)	1,129
Severe DDI	457 (82.05)	91 (16.34)	205 (36.80)	137 (24.60)	164 (29.44)	557
Moderate DDI	18 (2.25)	350 (43.75)	259 (32.38)	236 (29.50)	475 (59.38)	800
Minor DDI	0	191 (64.97)	106 (36.05)	31 (10.54)	22 (7.48)	294
CIT						
Potentially interacting drugs	426 (37.67)	691 (61.10)	582 (51.46)	392 (34.66)	706 (62.42)	1,131
Severe DDI	419 (77.59)	100 (18.52)	220 (40.74)	141 (26.11)	281 (52.04)	540
Moderate DDI	7 (0.85)	347 (42.37)	264 (32.23)	245 (29.91)	374 (45.67)	819
Minor DDI	0	244 (70.72)	98 (28.41)	6 (1.74)	51 (14.78)	345
PAR						
Potentially interacting drugs	457 (42.16)	635 (58.58)	515 (47.51)	305 (28.14)	492 (45.39)	1,084
Severe DDI	441 (84.16)	90 (17.18)	136 (25.95)	100 (19.08)	106 (20.23)	524
Moderate DDI	16 (2.12)	343 (45.37)	253 (33.47)	185 (24.47)	370 (48.94)	756
Minor DDI	0	202 (66.89)	126 (41.72)	20 (6.62)	16 (5.30)	302
SER						
Potentially interacting drugs	443 (36.73)	618 (51.24)	553 (45.85)	410 (34.00)	655 (54.31)	1,206
Severe DDI	430 (76.11)	57 (10.09)	198 (35.04)	158 (27.96)	158 (27.96)	565
Moderate DDI	13 (1.44)	365 (40.56)	241 (26.78)	226 (25.11)	493 (54.78)	900
Minor DDI	0	196 (66.89)	114 (38.91)	26 (8.87)	4 (1.37)	293
ESC						
Potentially interacting drugs	438 (38.22)	679 (59.25)	517 (45.11)	383 (33.42)	690 (60.21)	1,146
Severe DDI	431 (79.96)	87 (16.14)	133 (24.68)	137 (25.42)	264 (48.98)	539
Moderate DDI	7 (0.85)	359 (43.46)	270 (32.69)	214 (25.91)	378 (45.76)	826
Minor DDI	0	233 (63.32)	114 (30.98)	32 (8.70)	48 (13.04)	368

Abbreviations: SSRIs, selective serotonin reuptake inhibitors; ICs, Interaction checkers; DDIs, Drug-drug interactions; FXT, fluoxetine; FVM, fluvoxamine; CIT, citalopram; PAR = paroxetine; SER, sertraline; ESC, escitalopram. The total number represents the count of unique DDIs, identified across all five ICs, after removing duplicates.

**FIGURE 1:**
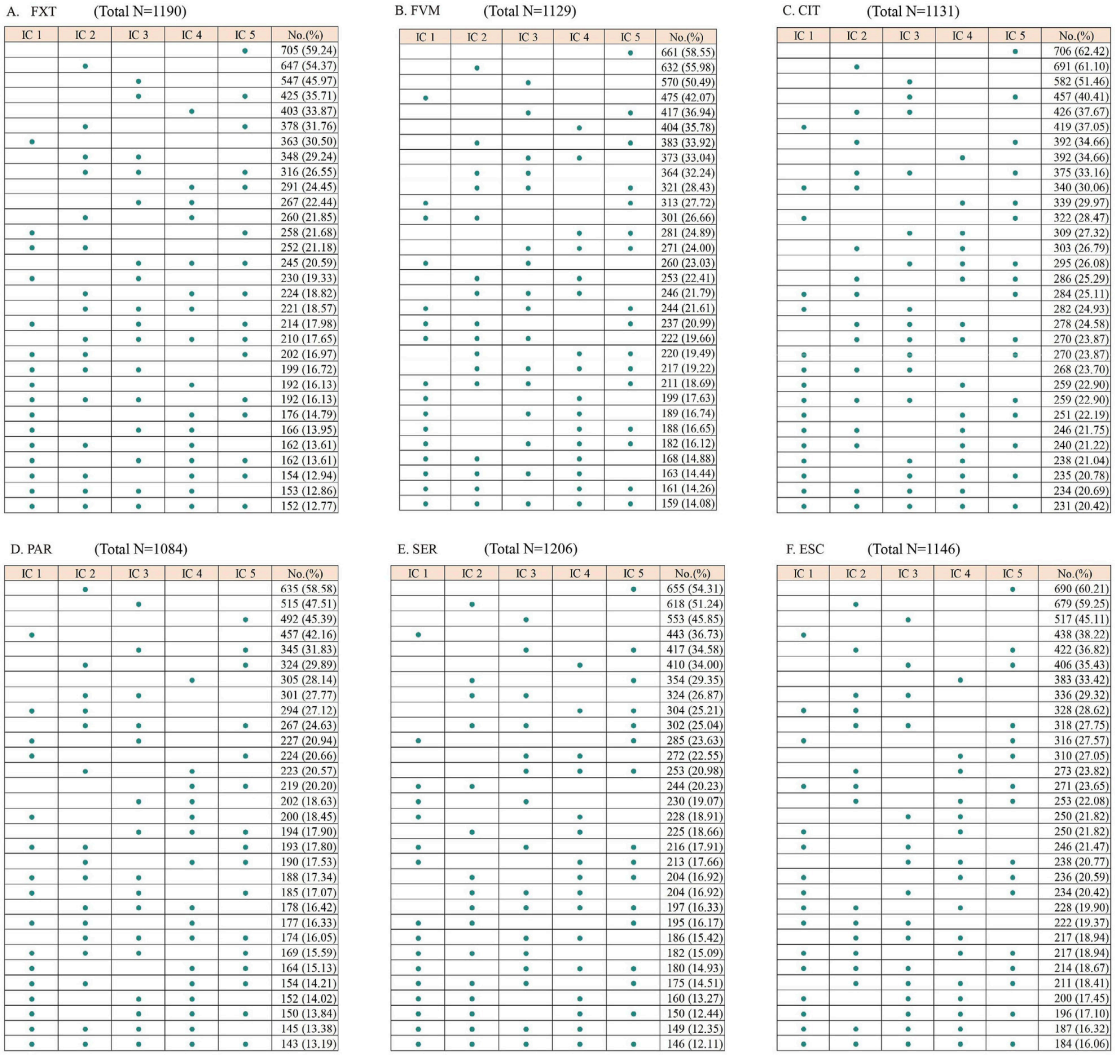
Matrices showing the number of drugs listed as potentially interacting with selective serotonin reuptake inhibitors by each interaction checker (IC). FXT, fluoxetine; FVM, fluvoxamine; CIT, citalopram; PAR, paroxetine; SER, sertraline; ESC, escitalopram. The total number represents the count of unique DDIs identified across all five ICs after removing duplicates.

**FIGURE 2:**
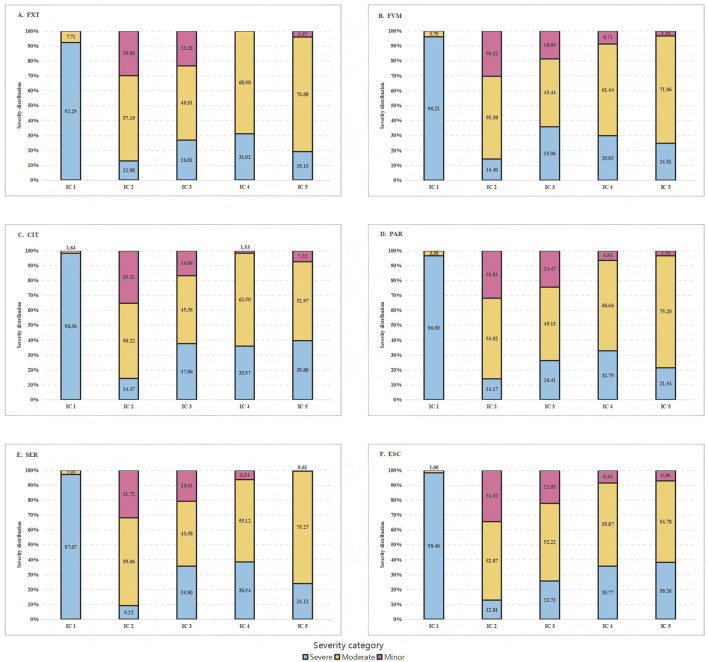
Severity category distribution by interaction checker (IC) of the six serotonin reuptake inhibitors. FXT, fluoxetine; FVM, fluvoxamine; CIT, citalopram; PAR, paroxetine; SER, sertraline; ESC, escitalopram.

Despite the wide range of reported interactions, only a small fraction was consistently flagged by all five ICs. Specifically, 12.77% of drugs were identified as interacting with FXT, 14.08% with FVM, 20.42% with CIT, 13.19% with PAR, 12.11% with SER, and 16.06% with ESC (Figure 1). There were significant differences in the classification of interaction severity among the ICs. IC 1 mostly categorized these as severe (92.29%–98.40%), while the other ICs predominantly classified them as moderate (43.58%–76.88%) (Table 1; Figure 2).

The overall agreement among the five different ICs in identifying potential DDIs was generally low, with agreement levels varying from 0.16 (95% CI, 0.14-0.18) for PAR to 0.24 (95% CI, 0.22-0.26) for CIT (Table 2). When examining combinations of four ICs, the Gwet AC1 scores varied greatly for different SSRIs evaluated, with the lowest score (0.10) observed in the FVM group without Epocrates, and the highest value (0.30) observed in the CIT group without Lexi-Interact (Supplementary Table S2). The exclusion of Lexi-Interact yielded the highest agreement in all six SSRIs, with Gwet AC1 statistic values ranging from 0.20 (95% CI, 0.18-0.23) for FXT to 0.30 (95% CI, 0.27-0.32) for CIT. In pairwise comparisons, Micromedex paired with Medscape showed a substantial agreement for FXT, CIT and ESC, with Gwet AC1 statistic values of 0.27, 0.43 and 0.39, respectively, while Epocrates and Drugs.com showed a substantial agreement for PAR and SER, with Gwet AC1 statistic values of 0.41 and 0.38, respectively. For FVM, Epocrates and Medscape showed the highest agreement in all pairwise comparisons, with Gwet AC1 statistic values of 0.59. Lexi-Interact and Drugs.com showed the poorest agreement for FXT, FVM, CIT and ESC, with Gwet AC1 statistic values ranging from −0.02 to 0.05. Lexi-Interact and Epocrates showed the poorest agreement for PAR, and Lexi-Interact and Medscape for SER, with Gwet AC1 statistic values of −0.02 and 0.02, respectively. Among the pairwise analysis, combinations involving Lexi-Interact demonstrated poorer agreement with other ICs in all six SSRIs. Notably, the Gwet AC1 scores between Lexi-Interact and Drugs.com for FXT and Lexi-Interact and Epocrates for PAR were negative, indicating either random agreement or notable disagreement.

**TABLE 2:** Agreement level in listing and severity categorization of potentially interacting drugs across 5 interaction checkers.

Category	Agreement (95% CI)^a^	*P* Value
FXT		
Interacting vs. noninteracting	0.16 (0.14, 0.19)	<0.001
All categories	0.13 (0.10, 0.15)	<0.001
Restricting to severe category	0.30 (0.28, 0.32)	<0.001
FVM		
Interacting vs. noninteracting	0.17 (0.15, 0.19)	<0.001
All categories	0.19 (0.13, 0.18)	<0.001
Restricting to severe category	0.27 (0.24, 0.29)	<0.001
CIT		
Interacting vs. noninteracting	0.24 (0.22, 0.26)	<0.001
All categories	0.18 (0.16, 0.20)	<0.001
Restricting to severe category	0.33 (0.31, 0.35)	<0.001
PAR		
Interacting vs. noninteracting	0.16 (0.14, 0.18)	<0.001
All categories	0.13 (0.10, 0.15)	<0.001
Restricting to severe category	0.22 (0.20, 0.25)	<0.001
SER		
Interacting vs. noninteracting	0.17 (0.15, 0.19)	<0.001
All categories	0.12 (0.10, 0.14)	<0.001
Restricting to severe category	0.23 (0.21, 0.25)	<0.001
ESC		
Interacting vs. noninteracting	0.20 (0.18, 0.23)	<0.001
All categories	0.14 (0.12, 0.17)	<0.001
Restricting to severe category	0.27 (0.25, 0.29)	<0.001

Abbreviations: FXT, fluoxetine; FVM, fluvoxamine; CIT, citalopram; PAR = paroxetine; SER, sertraline; ESC, escitalopram.

^a^ For Gwet AIC, 1 indicates perfect agreement; 0.76 to 1, excellent agreement; 0.41 to 0.75, intermediate to good agreement; 0 to 0.40, poor agreement; less than 0, disagreement; and −1, complete disagreement. Values around 0 with nonsignificant *P* values indicate agreement no different from chance.

When categorizing the severity of potential DDIs, the analysis yielded lower scores for all SSRIs under this study, with Gwet AC1 statistic values ranging from 0.12 (95% CI, 0.10-0.14) for SER to 0.18 (95% CI, 0.16-0.20) for CIT (Table 2). When restricting the analysis to the potential DDIs identified as severe by at least 1 IC, all SSRIs demonstrate a relatively higher agreement, with agreement levels varying from 0.22 (95% CI, 0.20-0.25) for PAR to 0.33 (95% CI, 0.31-0.35) for CIT (Table 2). The number of interactions simultaneously identified as severe by all ICs was minimal across SSRIs: 16 for FXT, 24 for FVM, 18 for CIT, 20 for PAR, 6 for SER, and 16 for ESC. The overall level of agreement was similar to previous results when comparing different groups of 4 ICs as well as different pairs of ICs, whether categorizing the severity of potential DDIs or restricting the severity as severe (Supplementary Table S3, S4). In general, in any of the three cases, the agreement was poor.

## 4 Discussion

To our knowledge, this is the first study to compare the effectiveness and agreement of five widely used DDI ICs in identifying interactions with SSRIs. The findings revealed considerable variability in both the identification of potential DDIs and their severity classifications among the ICs, accompanied by notably low agreement levels across the tools. The results highlighted the challenges that are faced by healthcare professionals when assessing the interaction risk and related safety of medication regimens, especially in populations at higher risk.

The discrepancies observed in the number of potential DDIs reported by different ICs highlight a critical variability in the tools’ performance. Drugs.com reported the highest number of potential DDIs across all SSRIs, followed by Epocrates, Medscape, Lexi-Interact, and Micromedex. Similarly, another descriptive analysis found that the IC reporting the highest number of potential DDIs was drugs.com, followed by Epocrates, Lexi-Interact, Micromedex, and INTERCheck WEB for all four direct oral anticoagulants (DOACs) included in the study (Carollo et al., 2024a). Another study also found Micromedex detected half the number obtained by the other two DDI programs (Medscape and Drugs.com) in detecting potential DDIs in a community pharmacy setting (Sancar et al., 2019). This disparity may stem from differences in the databases and algorithms employed by each IC, the frequency of updates, or the criteria used to define interactions. The higher reporting rate of Drugs.com suggests a more conservative approach or a broader database that captures more potential interactions, which could be advantageous in identifying possible risks. Even though there is a rise in the number of identified potential drug-drug interactions (DDIs), this does not necessarily enhance the accuracy or importance for clinicians during prescription decisions. In fact, it might distract from more crucial DDIs, making the decision-making process more challenging (Glassman et al., 2002; Page et al., 2017). Research by Pinkoh R et al. revealed that, unlike Epocrates and Lexi-Interact, Drugs.com identified over 130 times more psychotropic DDIs, yet none were deemed clinically significant (Pinkoh et al., 2023). Other studies have shown that between 49% and 96% of potential DDI alerts are either disregarded or overridden, which can lead to possible harm to patients (van der Sijs et al., 2006; Edrees et al., 2020).

The overall low agreement among the ICs, yielding Gwet’s AC1 values ranging from 0.159 to 0.242, raises concerns about the clinical reliability of these tools. For instance, levofloxacin was identified as interacting with FXT by Lexi-Interact and Medscape, whereas the other three ICs did not list this interaction. Our study also demonstrated a poor agreement among 5 ICs in classifying DDI severity, Gwet’s AC1 values ranging from 0.120 to 0.181. As an example, clarithromycin was identified as severely interacting with FVM by Micromedex, Epocrates and Medscape, whereas Drugs.com categorized this interaction as minor and as moderate in Lexi-Interact. Our findings are consistent with previous studies, although the classes of drugs, ICs and patient populations were not the same (Günay et al., 2022; Trifirò et al., 2006; Roblek et al., 2015; Vitry, 2007; Kontsioti et al., 2022; Shariff et al., 2021). A cross-sectional study found a large heterogeneity among Lexi-Interact, Micromedex, drugs.com, INTERCheck WEB and Epocrates in reporting information on potential DDIs with proton pump inhibitors (Gwet’s AC1 values ranged, 0.23-0.27) (Carollo et al., 2024b). Similarly, another study assessing the concordance of the same 5 ICs in detecting potential DDIs for oral anticoagulants also found a poor overall level of agreement (Gwet’s AC1 values ranged, 0.12-0.16) (Carollo et al., 2024a). A similarly low level of consensus was noted in classifying potentially serious DDIs, with Gwet’s AC1 values ranging from 0.30 to 0.32, highlighting the most crucial clinical data. For example, dihydroergotamine was identified as interacting with ESC by all ICs. However, while Epocrates and Drugs.com classified it as severe, the other 3 ICs classified it as moderate. Abarca J et al. found only 2.2% of major DDIs were listed in all four compendia (Drug Interaction Facts, Drug Interactions: Analysis and Management, Evaluations of Drug Interactions, and the MicroMedex DRUG-REAX program) (Abarca et al., 2024). Ekstein et al. also found the concordance rate was less than 30% even if severity levels were classified as high between programs (Ekstein et al., 2015).

Pairwise and group comparisons revealed substantial agreements between certain IC pairs, such as Micromedex and Medscape, which indicated potential areas for improving consistency among ICs. Another study found that Lexi-Interact and Epocrates had the strongest agreement for the psychotropic DDI identification (Pinkoh et al., 2023). The different drugs being studied may lead to variations in the consistency between the two different ICs. We also found that the exclusion of Lexi-Interact resulted in improved agreement scores across SSRIs, suggesting that Lexi-Interact’s performance may be less aligned with other ICs. This could be due to methodological differences, such as unique algorithms or criteria for interaction severity. Understanding these discrepancies can guide practitioners in selecting the most reliable IC for their needs.

Notably, the overall agreement level was higher (Gwet’s AC1 values range, 0.22-0.24) compared to when the analysis was limited to groups of four ICs (Gwet’s AC1 values range, 0.10 -0.20) or to pairwise comparisons (Gwet’s AC1 values range, −0.02-0.59) in some cases. This increase in agreement with more ICs could result from a greater likelihood of them coincidentally identifying the same interactions. Furthermore, certain ICs might naturally align with one another, offsetting the disagreements of other ICs. When comparing all five ICs together, these compensatory agreements could enhance the overall agreement metric (Gwet, 2014; Hallgren, 2012).

The heterogeneity among different ICs may be attributed to several factors. Vitry stated the reasons for this discordance between ICs as various inclusion criteria, different information sources, and dissimilar therapeutic drug classifications in each program used, and also the severity classification based on the clinical relevance of each DDI was not common between different ICs (Vitry, 2007). Ekstein et al. also stated the discrepancies could be attributed to differences in definitions and terminology in each program, various clarifications of information in the literature, and different classifications of drugs used in various DDI programs (Ekstein et al., 2015). The classification of severity levels given by various ICs to potential DDIs often fails to accurately represent their actual clinical implications (Suriyapakorn et al., 2019). This inconsistency can be attributed to multiple factors, such as the distinct algorithms each IC employs to identify possible DDIs, the specific data sources they utilize, and the frequency with which these tools undergo updates.

Our findings indicated that the ICs often disagree on identifying potential DDIs. These discrepancies have significant implications for clinical decision-making. Healthcare providers rely on these tools to assess the safety of concurrent drug use, and variability in DDI identification can lead to inconsistent prescribing practices. Clinicians may experience uncertainty when different ICs provide conflicting information, potentially affecting their confidence in the reliability of these tools. The differences in severity classifications among the ICs further complicate clinical outcomes. The tendency of Micromedex to classify DDIs as severe, in contrast to the moderate classifications by other ICs, could influence prescriber behaviour significantly. Severity ratings inform the urgency and extent of interventions required, and discrepancies in these ratings could lead to either overestimation or underestimation of risks. The clinical relevance of severity classifications is paramount; prescribers may react differently based on the perceived severity, impacting patient safety and treatment efficacy. The number of potential DDIs simultaneously identified as severe by 5 ICs was very low in our study. Given such a low level of consistency, the clinicians should check more than one drug interaction database in clinical practice (Monteith and Glenn, 2019). Our findings also underscore the need for standardization and harmonization among ICs to improve their reliability and utility in clinical practice. Standardization efforts could include regular updates to IC databases, consensus on severity categorizations, and the adoption of uniform criteria for interaction identification. Such measures would enhance the consistency and reliability of ICs, supporting safer prescribing practices and reducing the burden on healthcare providers. These recommendations could ensure that DDI checkers serve as effective tools in optimizing patient safety and treatment outcomes.

This study systematacially evaluated the identification and severity of DDIs as documented in 5 globally utilized ICs and compared ICs using the Gwet AC1 statistic, yielding more reliable results in the presence of significant imbalances in the number of total drugs identified and in severity categories compared to Fleiss’ kappa and Cohen’s kappa (McHugh, 2012; Popplewell et al., 2019).

However, there are limitations to this study. First, no updates have been taken into consideration since the date of data retrieval. Consequently, findings characterize the similarity and consistency status at that temporal snapshot, notwithstanding the infrequency of major revisions. Second, we were not able to evaluate the clinical relevance of SSRI-related DDIs because all five IC databases used integrate preclinical and clinical data, potentially overestimating clinical risk by including theoretical interactions and increasing inconsistencies between different IC databases. Third, ICs typically assess only individual drug interactions, disregarding possible cumulative or synergistic effects from multiple interactions. This is particularly significant for elderly patients or other patients who often take multiple medications. Fourth, recognizing the lack of DDIs is as important as acknowledging their presence or severity. However, ICs typically only list interacting drugs, omitting non-interacting ones. With many drugs unmentioned, statistical concordance assessments on DDI absence may misleadingly suggest high agreement, failing to reflect ICs’ true reliability in detecting clinically significant DDIs. Fifth, the consistency among any three databases was not analyzed. Our primary consideration was that 3-set analysis increased the reporting burden without significantly enhancing conclusions, particularly when the 4-set and 2-set already demonstrate low consistency. It may limit insights into consistency for 3 ICs use, even as 2/4/5-IC results showed low consistency and underscored IC standardization needs. Lastly, all 5 databases used static qualitative interaction classifications without accounting for drug exposure variations, and the lack of dose-dependency considerations limited their clinical applicability. Additionally, we did not consider interactions with dietary supplements, commonly excluded from some ICs.

Several workgroups have developed specific recommendations to improve the quality of clinical decision support (CDS) alerts for DDIs or to create a process to establish a standard set of DDIs for CDS. The key work is to evaluate DDI evidence from various ICs and make recommendations as to what interactions should be included in CDS systems (Tilson et al., 2016; Scheife et al., 2015; Payne et al., 2015). Future research should explore the integration of artificial intelligence (AI) technologies combined with evaluating DDI evidence. AI could potentially analyze vast amounts of data more efficiently and consistently, leading to improved DDI identification and severity classification.

## 5 Conclusion

Our study highlighted the discrepancies among different ICs in the identification and severity categories of DDIs for SSRIs. The need for standardization and update of the IC databases, consensus on severity categorizations, the adoption of uniform criteria for interaction identification, and creating a process to establish a standard set of DDIs is urgent. More real-world studies could facilitate the development of a gold-standard DDI dataset.

## Data Availability

The original contributions presented in the study are included in the article/Supplementary Material, further inquiries can be directed to the corresponding author.
