# Supplementary material for: A comparison of five different drug-drug interaction checkers for selective serotonin reuptake inhibitors
Source: Front Pharmacol. 2025 Sep 24;16:1690975. doi: 10.3389/fphar.2025.1690975 (PMC12504084; doi:10.3389/fphar.2025.1690975)
Supplement: Supplementary file 1 [file Supplementaryfile1.docx]

**Supplemental materials**

**Supplemental Table 1. Reclassification of DDI severity categories as defined by the 5 drug interaction checkers**

|  | **DDI severity category as indicated by drug interaction checkers** | | | | |  |
| --- | --- | --- | --- | --- | --- | --- |
| **Reclassification of DDI severity category** | **Micromedex** | **Lexi-interact** | \| **Epocrates** \| \| --- \| | **Medscape** | **Drugs.com** | |
| Severe | Contraindicated  Major | X. Avoid combination  D. Consider therapy modification | Contraindicated  Avoid/Use Alternative | ContraindicatedSerious | Major | |
| Moderate | Moderate | C. Monitor therapy | Monitor/Modify Therapy | Monitor Closely | Moderate | |
| Minor | Minor | B. No action needed | Caution advised | Minor | Minor | |
| Unknown | - | No known interaction | - | - | Unknown | |

Abbreviations: DDI = Drug-drug interaction

**Supplemental Table 2. Level of agreement in listing DDIs among groups of 4 ICs and different pairs of ICs, using the Gwet AC1 statistic**

| Category | Agreement (95% CI) | *P* value |
| --- | --- | --- |
| **FXT** |  |  |
| **Comparison of groups of 4 ICs** |  |  |
| Without Micromedex | 0.15 (0.09, 0.14) | < 0.001 |
| Without Lexicomp | 0.20 (0.18, 0.23) | < 0.001 |
| Without Epocrates | 0.12 (0.10, 0.15) | < 0.001 |
| Without Medscape | 0.14 (0.11, 0.17) | < 0.001 |
| Without Drugs.com | 0.18 (0.16, 0.21) | < 0.001 |
| **Pairwise comparison of ICs** |  |  |
| Micromedex - Lexicomp | 0.13 (0.09, 0.19) | < 0.001 |
| Micromedex - Epocrates | 0.17 (0.13, 0.21) | < 0.001 |
| Micromedex - Medscape | 0.27 (0.23, 0.30) | < 0.001 |
| Micromedex - Drugs.com | 0.06 (0.02, 0.10) | 0.002 |
| Lexicomp - Epocrates | 0.13 (0.09, 0.17) | < 0.001 |
| Lexicomp - Medscape | 0.10 (0.06, 0.14) | < 0.001 |
| Lexicomp - Drugs.com | -0.02 (-0.06, 0.10) | 0.301 |
| Epocrates - Medscape | 0.24 (0.21, 0.28) | < 0.001 |
| Epocrates - Drugs.com | 0.29 (0.25, 0.32) | < 0.001 |
| Medscape - Drugs.com | 0.11 (0.07, 0.15) | < 0.001 |
| **FVM** |  |  |
| **Comparison of groups of 4 ICs** |  |  |
| Without Micromedex | 0.22 (0.19, 0.24) | < 0.001 |
| Without Lexicomp | 0.22 (0.19, 0.24) | < 0.001 |
| Without Epocrates | 0.10 (0.07, 0.12) | < 0.001 |
| Without Medscape | 0.13 (0.10, 0.16) | < 0.001 |
| Without Drugs.com | 0.19 (0.16, 0.21) | < 0.001 |
| **Pairwise comparison of ICs** |  |  |
| Micromedex - Lexicomp | 0.11 (0.07, 0.14) | < 0.001 |
| Micromedex - Epocrates | 0.07 (0.03, 0.11) | 0.001 |
| Micromedex - Medscape | 0.10 (0.07, 0.14) | < 0.001 |
| Micromedex - Drugs.com | 0.10 (0.06, 0.14) | < 0.001 |
| Lexicomp - Epocrates | 0.16 (0.12, 0.20) | < 0.001 |
| Lexicomp - Medscape | 0.06 (0.02, 0.10) | < 0.001 |
| Lexicomp - Drugs.com | 0.05 (0.01, 0.09) | 0.008 |
| Epocrates - Medscape | 0.59 (0.56, 0.62) | 0.024 |
| Epocrates - Drugs.com | 0.29 (0.26, 0.33) | < 0.001 |
| Medscape - Drugs.com | 0.11 (0.07, 0.15) | < 0.001 |
| **CIT** |  |  |
| **Comparison of groups of 4 ICs** |  |  |
| Without Micromedex | 0.23 (0.20, 0.25) | < 0.001 |
| Without Lexicomp | 0.30 (0.27, 0.32) | < 0.001 |
| Without Epocrates | 0.22 (0.20, 0.25) | < 0.001 |
| Without Medscape | 0.19 (0.16, 0.22) | < 0.001 |
| Without Drugs.com | 0.26 (0.24, 0.29) | < 0.001 |
| **Pairwise comparison of ICs** |  |  |
| Micromedex - Lexicomp | 0.23 (0.19, 0.26) | < 0.001 |
| Micromedex - Epocrates | 0.21 (0.17, 0.24) | < 0.001 |
| Micromedex - Medscape | 0.43 (0.39, 0.46) | < 0.001 |
| Micromedex - Drugs.com | 0.14 (0.10, 0.18) | < 0.001 |
| Lexicomp - Epocrates | 0.14 (0.11, 0.18) | < 0.001 |
| Lexicomp - Medscape | 0.16 (0.12, 0.19) | < 0.001 |
| Lexicomp - Drugs.com | 0.04 (0.00, 0.08) | 0.081 |
| Epocrates - Medscape | 0.36 (0.33, 0.39) | < 0.001 |
| Epocrates - Drugs.com | 0.33 (0.29, 0.36) | < 0.001 |
| Medscape - Drugs.com | 0.26 (0.22, 0.29) | < 0.001 |
| **PAR** |  |  |
| **Comparison of groups of 4 ICs** |  |  |
| Without Micromedex | 0.19 (0.16, 0.22) | < 0.001 |
| Without Lexicomp | 0.21 (0.19, 0.24) | < 0.001 |
| Without Epocrates | 0.16 (0.10, 0.18) | < 0.001 |
| Without Medscape | 0.12 (0.09, 0.15) | < 0.001 |
| Without Drugs.com | 0.11 (0.09, 0.14) | < 0.001 |
| **Pairwise comparison of ICs** |  |  |
| Micromedex - Lexicomp | 0.07 (0.03, 0.11) | 0.001 |
| Micromedex - Epocrates | 0.03 (-0.01, 0.08) | 0.108 |
| Micromedex - Medscape | 0.27 (0.23, 0.30) | < 0.001 |
| Micromedex - Drugs.com | 0.06 (0.02, 0.10) | 0.003 |
| Lexicomp - Epocrates | -0.02 (-0.06, 0.03) | 0.493 |
| Lexicomp - Medscape | 0.07 (0.03, 0.11) | < 0.001 |
| Lexicomp - Drugs.com | 0.12 (0.08, 0.15) | < 0.001 |
| Epocrates - Medscape | 0.18 (0.15, 0.22) | < 0.001 |
| Epocrates - Drugs.com | 0.41 (0.38, 0.44) | < 0.001 |
| Medscape - Drugs.com | 0.29 (0.25, 0.32) | < 0.001 |
| **SER** |  |  |
| **Comparison of groups of 4 ICs** |  |  |
| Without Micromedex | 0.19 (0.16, 0.22) | < 0.001 |
| Without Lexicomp | 0.23 (0.21, 0.26) | < 0.001 |
| Without Epocrates | 0.13 (0.11, 0.16) | < 0.001 |
| Without Medscape | 0.14 (0.11, 0.17) | < 0.001 |
| Without Drugs.com | 0.14 (0.16, 0.17) | < 0.001 |
| **Pairwise comparison of ICs** |  |  |
| Micromedex - Lexicomp | 0.04 (0.00, 0.08) | 0.073 |
| Micromedex - Epocrates | 0.08 (0.05, 0.12) | < 0.001 |
| Micromedex - Medscape | 0.28 (0.25, 0.31) | < 0.001 |
| Micromedex - Drugs.com | 0.12 (0.08, 0.16) | < 0.001 |
| Lexicomp - Epocrates | 0.13 (0.10, 0.17) | < 0.001 |
| Lexicomp - Medscape | 0.02 (-0.02, 0.06) | 0.318 |
| Lexicomp - Drugs.com | 0.06 (0.02, 0.10) | 0.002 |
| Epocrates - Medscape | 0.28 (0.24, 0.31) | < 0.001 |
| Epocrates - Drugs.com | 0.38 (0.35, 0.41) | < 0.001 |
| Medscape - Drugs.com | 0.23 (0.20, 0.27) | < 0.001 |
| **ESC** |  |  |
| **Comparison of groups of 4 ICs** |  |  |
| Without Micromedex | 0.18 (0.16, 0.21) | < 0.001 |
| Without Lexicomp | 0.26 (0.23, 0.28) | < 0.001 |
| Without Epocrates | 0.19 (0.17, 0.22) | < 0.001 |
| Without Medscape | 0.17 (0.14, 0.19) | < 0.001 |
| Without Drugs.com | 0.21 (0.18, 0.23) | < 0.001 |
| **Pairwise comparison of ICs** |  |  |
| Micromedex - Lexicomp | 0.20 (0.16, 0.23) | < 0.001 |
| Micromedex - Epocrates | 0.17 (0.13, 0.21) | < 0.001 |
| Micromedex - Medscape | 0.39 (0.36, 0.43) | < 0.001 |
| Micromedex - Drugs.com | 0.13 (0.10, 0.17) | < 0.001 |
| Lexicomp - Epocrates | 0.08 (0.05, 0.12) | < 0.001 |
| Lexicomp - Medscape | 0.10 (0.06, 0.13) | < 0.001 |
| Lexicomp - Drugs.com | 0.05 (0.01, 0.09) | 0.019 |
| Epocrates - Medscape | 0.27 (0.23, 0.30) | < 0.001 |
| Epocrates - Drugs.com | 0.31 (0.28, 0.34) | < 0.001 |
| Medscape - Drugs.com | 0.21 (0.17, 0.24) | < 0.001 |

**Supplemental Table 3. Level of agreement in categorizing the DDI severity among groups of 4 ICs and different pairs of ICs, using the Gwet AC1 statistic**

| Category | Agreement (95% CI) | *P* value |
| --- | --- | --- |
| **FXT** |  |  |
| **Comparison of groups of 4 ICs** |  |  |
| Without Micromedex | 0.12 (0.09, 0.14) | < 0.001 |
| Without Lexicomp | 0.18 (0.15, 0.20) | < 0.001 |
| Without Epocrates | 0.09 (0.06, 0.12) | < 0.001 |
| Without Medscape | 0.11 (0.08, 0.14) | < 0.001 |
| Without Drugs.com | 0.13 (0.10, 0.16) | < 0.001 |
| **Pairwise comparison of ICs** |  |  |
| Micromedex - Lexicomp | 0.05 (0.01, 0.09) | 0.01 |
| Micromedex - Epocrates | 0.16 (0.12, 0.20) | < 0.001 |
| Micromedex - Medscape | 0.17 (0.13, 0.20) | < 0.001 |
| Micromedex - Drugs.com | 0.04 (0.00, 0.08) | 0.046 |
| Lexicomp - Epocrates | 0.08 (0.04, 0.12) | < 0.001 |
| Lexicomp - Medscape | 0.05 (0.01, 0.09) | 0.014 |
| Lexicomp - Drugs.com | -0.05 (-0.09, -0.01) | 0.019 |
| Epocrates - Medscape | 0.19 (0.16, 0.29) | < 0.001 |
| Epocrates - Drugs.com | 0.24 (0.20, 0.27) | < 0.001 |
| Medscape - Drugs.com | 0.14 (0.11, 0.18) | < 0.001 |
| **FVM** |  |  |
| **Comparison of groups of 4 ICs** |  |  |
| Without Micromedex | 0.19 (0.17, 0.22) | < 0.001 |
| Without Lexicomp | 0.23 (0.20, 0.25) | < 0.001 |
| Without Epocrates | 0.08 (0.05, 0.11) | < 0.001 |
| Without Medscape | 0.11 (0.08, 0.13) | < 0.001 |
| Without Drugs.com | 0.17 (0.15, 0.20) | < 0.001 |
| **Pairwise comparison of ICs** |  |  |
| Micromedex - Lexicomp | 0.01 (-0.03, 0.05) | 0.553 |
| Micromedex - Epocrates | 0.100 (0.06, 0.14) | < 0.001 |
| Micromedex - Medscape | 0.088 (0.05, 0.13) | < 0.001 |
| Micromedex - Drugs.com | 0.041 (0.00, 0.08) | 0.048 |
| Lexicomp - Epocrates | 0.104 (0.07, 0.14) | < 0.001 |
| Lexicomp - Medscape | 0.036 (-0.01, 0.08) | 0.086 |
| Lexicomp - Drugs.com | 0.00 (-0.04, 0.04) | 0.881 |
| Epocrates - Medscape | 0.61 (0.58, 0.63) | < 0.001 |
| Epocrates - Drugs.com | 0.25 (0.22, 0.29) | < 0.001 |
| Medscape - Drugs.com | 0.15 (0.11, 0.19) | < 0.001 |
| **CIT** |  |  |
| **Comparison of groups of 4 ICs** |  |  |
| Without Micromedex | 0.17 (0.14, 0.19) | < 0.001 |
| Without Lexicomp | 0.26 (0.24, 0.29) | < 0.001 |
| Without Epocrates | 0.15 (0.12, 0.17) | < 0.001 |
| Without Medscape | 0.15 (0.12, 0.17) | < 0.001 |
| Without Drugs.com | 0.17 (0.15, 0.20) | < 0.001 |
| **Pairwise comparison of ICs** |  |  |
| Micromedex - Lexicomp | 0.07 (0.03, 0.11) | < 0.001 |
| Micromedex - Epocrates | 0.19 (0.15, 0.23) | < 0.001 |
| Micromedex - Medscape | 0.25 (0.22, 0.29) | < 0.001 |
| Micromedex - Drugs.com | 0.17 (0.13, 0.20) | < 0.001 |
| Lexicomp - Epocrates | 0.06 (0.02, 0.10) | 0.006 |
| Lexicomp - Medscape | 0.07 (0.03, 0.11) | 0.001 |
| Lexicomp - Drugs.com | -0.02 (-0.06, 0.03) | 0.422 |
| Epocrates - Medscape | 0.32 (0.9, 0.36) | < 0.001 |
| Epocrates - Drugs.com | 0.31 (0.28, 0.34) | < 0.001 |
| Medscape - Drugs.com | 0.23 (0.20, 0.27) | < 0.001 |
| **PAR** |  |  |
| **Comparison of groups of 4 ICs** |  |  |
| Without Micromedex | 0.15 (0.13, 0.18) | < 0.001 |
| Without Lexicomp | 0.17 (0.14, 0.20) | < 0.001 |
| Without Epocrates | 0.11 (0.08, 0.14) | < 0.001 |
| Without Medscape | 0.10 (0.07, 0.12) | < 0.001 |
| Without Drugs.com | 0.09 (0.06, 0.12) | < 0.001 |
| **Pairwise comparison of ICs** |  |  |
| Micromedex - Lexicomp | 0.00 (-0.04, 0.04) | 0.939 |
| Micromedex - Epocrates | 0.03 (-0.01, 0.07) | 0.172 |
| Micromedex - Medscape | 0.14 (0.11, 0.18) | < 0.001 |
| Micromedex - Drugs.com | 0.01 (-0.03, 0.05) | 0.667 |
| Lexicomp - Epocrates | 0.02 (-0.03, 0.06) | 0.420 |
| Lexicomp - Medscape | 0.06 (0.02, 0.10) | 0.006 |
| Lexicomp - Drugs.com | 0.08 (0.04, 0.12) | < 0.001 |
| Epocrates - Medscape | 0.16 (0.13, 0.20) | < 0.001 |
| Epocrates - Drugs.com | 0.32 (0.29, 0.35) | < 0.001 |
| Medscape - Drugs.com | 0.25 (0.21, 0.29) | < 0.001 |
| **SER** |  |  |
| **Comparison of groups of 4 ICs** |  |  |
| Without Micromedex | 0.14 (0.11, 0.16) | < 0.001 |
| Without Lexicomp | 0.19 (0.17, 0.22) | < 0.001 |
| Without Epocrates | 0.07 (0.05, 0.10) | < 0.001 |
| Without Medscape | 0.091 (0.06, 0.12) | < 0.001 |
| Without Drugs.com | 0.10 (0.07, 0.12) | < 0.001 |
| **Pairwise comparison of ICs** |  |  |
| Micromedex - Lexicomp | -0.05 (-0.09, -0.01) | 0.019 |
| Micromedex - Epocrates | 0.11 (0.07, 0.14) | < 0.001 |
| Micromedex - Medscape | 0.15 (0.19, 0.19) | < 0.001 |
| Micromedex - Drugs.com | 0.036 (0.00, 0.08) | 0.069 |
| Lexicomp - Epocrates | 0.06 (0.02, 0.09) | 0.005 |
| Lexicomp - Medscape | -0.001 (-0.05, 0.03) | 0.719 |
| Lexicomp - Drugs.com | -0.03 (-0.08, 0.01) | 0.096 |
| Epocrates - Medscape | 0.24 (0.21, 0.28) | < 0.001 |
| Epocrates - Drugs.com | 0.30 (0.27 0.34) | < 0.001 |
| Medscape - Drugs.com | 0.21 (0.17, 0.25) | < 0.001 |
| **ESC** |  |  |
| **Comparison of groups of 4 ICs** |  |  |
| Without Micromedex | 0.13 (0.10, 0.16) | < 0.001 |
| Without Lexicomp | 0.21 (0.18, 0.23) | < 0.001 |
| Without Epocrates | 0.13 (0.10, 0.16) | < 0.001 |
| Without Medscape | 0.11 (0.08, 0.14) | < 0.001 |
| Without Drugs.com | 0.13 (0.10, 0.16) | < 0.001 |
| **Pairwise comparison of ICs** |  |  |
| Micromedex - Lexicomp | 0.04 (0.00, 0.08) | 0.065 |
| Micromedex - Epocrates | 0.10 (0.06, 0.14) | < 0.001 |
| Micromedex - Medscape | 0.24 (0.20, 0.27) | < 0.001 |
| Micromedex - Drugs.com | 0.16 (0.12, 0.20) | < 0.001 |
| Lexicomp - Epocrates | 0.05 (0.01, 0.09) | 0.012 |
| Lexicomp - Medscape | 0.05 (0.01, 0.09) | 0.024 |
| Lexicomp - Drugs.com | -0.02 (-0.06, 0.02) | 0.359 |
| Epocrates - Medscape | 0.21 (0.18, 0.25) | < 0.001 |
| Epocrates - Drugs.com | 0.22 (0.19, 0.26) | < 0.001 |
| Medscape - Drugs.com | 0.22 (0.18, 0.25) | < 0.001 |

**Supplemental Table 4. Level of agreement in categorizing DDIs as severe among groups of 4 ICs and different pairs of ICs, using the Gwet AC1 statistic**

| Category | Agreement (95% CI) | *P* value |
| --- | --- | --- |
| **FXT** |  |  |
| **Comparison of groups of 4 ICs** |  |  |
| Without Micromedex | 0.32 (0.30, 0.34) | < 0.001 |
| Without Lexicomp | 0.36 (0.34, 0.38) | < 0.001 |
| Without Epocrates | 0.25 (0.23, 0.28) | < 0.001 |
| Without Medscape | 0.30 (0.27, 0.32) | < 0.001 |
| Without Drugs.com | 0.25 (0.23, 0.28) | < 0.001 |
| **Pairwise comparison of ICs** |  |  |
| Micromedex - Lexicomp | 0.17 (0.14, 0.21) | < 0.001 |
| Micromedex - Epocrates | 0.35 (0.32, 0.38) | < 0.001 |
| Micromedex - Medscape | 0.20 (0.16, 0.23) | < 0.001 |
| Micromedex - Drugs.com | 0.30 (0.26, 0.33) | < 0.001 |
| Lexicomp - Epocrates | 0.13 (0.09, 0.16) | < 0.001 |
| Lexicomp - Medscape | 0.15 (0.11, 0.18) | < 0.001 |
| Lexicomp - Drugs.com | 0.16 (0.12, 0.20) | < 0.001 |
| Epocrates - Medscape | 0.38 (0.35, 0.42) | < 0.001 |
| Epocrates - Drugs.com | 0.54 (0.52, 0.57) | < 0.001 |
| Medscape - Drugs.com | 0.46 (0.43, 0.49) | < 0.001 |
| **FVM** |  |  |
| **Comparison of groups of 4 ICs** |  |  |
| Without Micromedex | 0.30 (0.28, 0.32) | < 0.001 |
| Without Lexicomp | 0.33 (0.29, 0.37) | < 0.001 |
| Without Epocrates | 0.21 (0.16, 0.26) | < 0.001 |
| Without Medscape | 0.23 (0.19, 0.28) | < 0.001 |
| Without Drugs.com | 0.22 (0.17, 0.27) | < 0.001 |
| **Pairwise comparison of ICs** |  |  |
| Micromedex - Lexicomp | 0.05 (-0.03, 0.12) | 0.236 |
| Micromedex - Epocrates | 0.26 (0.19, 0.32) | < 0.001 |
| Micromedex - Medscape | 0.15 (0.08, 0.22) | < 0.001 |
| Micromedex - Drugs.com | 0.20 (0.13, 0.27) | < 0.001 |
| Lexicomp - Epocrates | 0.08 (-0.03, 0.18) | 0.161 |
| Lexicomp - Medscape | 0.20 (0.09, 0.31) | 0.000 |
| Lexicomp - Drugs.com | 0.16 (0.05, 0.27) | 0.005 |
| Epocrates - Medscape | 0.47 (0.39, 0.55) | < 0.001 |
| Epocrates - Drugs.com | 0.53 (0.46, 0.60) | < 0.001 |
| Medscape - Drugs.com | 0.43 (0.34, 0.51) | < 0.001 |
| **CIT** |  |  |
| **Comparison of groups of 4 ICs** |  |  |
| Without Micromedex | 0.33 (0.30, 0.35) | < 0.001 |
| Without Lexicomp | 0.43 (0.40, 0.45) | < 0.001 |
| Without Epocrates | 0.27 (0.25, 0.30) | < 0.001 |
| Without Medscape | 0.33 (0.30, 0.35) | < 0.001 |
| Without Drugs.com | 0.27 (0.25, 0.30) | < 0.001 |
| **Pairwise comparison of ICs** |  |  |
| Micromedex - Lexicomp | 0.13 (0.09, 0.17) | < 0.001 |
| Micromedex - Epocrates | 0.39 (0.36, 0.42) | < 0.001 |
| Micromedex - Medscape | 0.23 (0.19, 0.26) | < 0.001 |
| Micromedex - Drugs.com | 0.45 (0.42, 0.48) | < 0.001 |
| Lexicomp - Epocrates | 0.11 (0.07, 0.14) | < 0.001 |
| Lexicomp - Medscape | 0.09 (0.05, 0.13) | < 0.001 |
| Lexicomp - Drugs.com | 0.10 (0.06, 0.14) | < 0.001 |
| Epocrates - Medscape | 0.51 (0.48, 0.54) | < 0.001 |
| Epocrates - Drugs.com | 0.56 (0.53, 0.59) | < 0.001 |
| Medscape - Drugs.com | 0.41 (0.37, 0.44) | < 0.001 |
| **PAR** |  |  |
| **Comparison of groups of 4 ICs** |  |  |
| Without Micromedex | 0.34 (0.32, 0.37) | < 0.001 |
| Without Lexicomp | 0.25 (0.22, 0.27) | < 0.001 |
| Without Epocrates | 0.18 (0.15, 0.20) | < 0.001 |
| Without Medscape | 0.19 (0.16, 0.21) | < 0.001 |
| Without Drugs.com | 0.17 (0.14, 0.20) | < 0.001 |
| **Pairwise comparison of ICs** |  |  |
| Micromedex - Lexicomp | 0.04 (0.00, 0.08) | 0.059 |
| Micromedex - Epocrates | 0.11 (0.08, 0.15) | < 0.001 |
| Micromedex - Medscape | 0.08 (0.04, 0.12) | < 0.001 |
| Micromedex - Drugs.com | 0.07 (0.03, 0.11) | 0.001 |
| Lexicomp - Epocrates | 0.14 (0.10, 0.18) | < 0.001 |
| Lexicomp - Medscape | 0.20 (0.17, 0.24) | < 0.001 |
| Lexicomp - Drugs.com | 0.24 (0.20, 0.27) | < 0.001 |
| Epocrates - Medscape | 0.41 (0.38, 0.44) | < 0.001 |
| Epocrates - Drugs.com | 0.54 (0.52, 0.57) | < 0.001 |
| Medscape - Drugs.com | 0.47 (0.44, 0.51) | < 0.001 |
| **SER** |  |  |
| **Comparison of groups of 4 ICs** |  |  |
| Without Micromedex | 0.29 (0.26, 0.31) | < 0.001 |
| Without Lexicomp | 0.32 (0.30, 0.35) | < 0.001 |
| Without Epocrates | 0.15 (0.13, 0.18) | < 0.001 |
| Without Medscape | 0.20 (0.17, 0.23) | < 0.001 |
| Without Drugs.com | 0.17 (0.14, 0.20) | < 0.001 |
| **Pairwise comparison of ICs** |  |  |
| Micromedex - Lexicomp | -0.05 (-0.09, -0.01) | 0.012 |
| Micromedex - Epocrates | 0.27 (0.23, 0.30) | < 0.001 |
| Micromedex - Medscape | 0.13 (0.09, 0.17) | < 0.001 |
| Micromedex - Drugs.com | 0.19 (0.15, 0.22) | < 0.001 |
| Lexicomp - Epocrates | -0.02 (-0.06, 0.02) | 0.291 |
| Lexicomp - Medscape | 0.00 (-0.04, 0.04) | 0.834 |
| Lexicomp - Drugs.com | 0.00 (-0.04, 0.04) | 0.834 |
| Epocrates - Medscape | 0.446 (0.42, 0.48) | < 0.001 |
| Epocrates - Drugs.com | 0.57 (0.54, 0.59) | < 0.001 |
| Medscape - Drugs.com | 0.43 (0.40, 0.46) | < 0.001 |
| **ESC** |  |  |
| **Comparison of groups of 4 ICs** |  |  |
| Without Micromedex | 0.28 (0.26, 0.30) | < 0.001 |
| Without Lexicomp | 0.34 (0.32, 0.37) | < 0.001 |
| Without Epocrates | 0.27 (0.24, 0.29) | < 0.001 |
| Without Medscape | 0.23 (0.21, 0.26) | < 0.001 |
| Without Drugs.com | 0.21 (0.18, 0.23) | < 0.001 |
| **Pairwise comparison of ICs** |  |  |
| Micromedex - Lexicomp | 0.06 (0.02, 0.10) | 0.002 |
| Micromedex - Epocrates | 0.17 (0.13, 0.21) | < 0.001 |
| Micromedex - Medscape | 0.21 (0.18, 0.25) | < 0.001 |
| Micromedex - Drugs.com | 0.42 (0.39, 0.46) | < 0.001 |
| Lexicomp - Epocrates | 0.09 (0.05, 0.12) | < 0.001 |
| Lexicomp - Medscape | 0.17 (0.13, 0.21) | < 0.001 |
| Lexicomp - Drugs.com | 0.06 (0.02, 0.10) | 0.002 |
| Epocrates - Medscape | 0.41 (0.38, 0.44) | < 0.001 |
| Epocrates - Drugs.com | 0.34 (0.31, 0.37) | < 0.001 |
| Medscape - Drugs.com | 0.47 (0.44, 0.50) | < 0.001 |

Abbreviations: CI = Confidence Interval, ICs = Interaction checkers; DDIs = Drug-drug interactions; FXT = fluoxetine, FVM = fluvoxamine, CIT = citalopram, PAR = paroxetine, SER = sertraline; ESC = escitalopram

Legend for Gwet’s AC1 coefficient interpretation: 

+1: Perfect Agreement 

0.76 to 1: Excellent Agreement 

0.41 to 0.75: Intermediate to Good Agreement 

0 to 0.40: Poor Agreement 

Less than 0: Disagreement 

-1: Complete Disagreement

Note: Values around zero with a non-significant p-value indicate agreement no different from chance.
